# Notes and News

**Published:** 1898-11-19

**Authors:** 


### NOTES AND NEWS.

(COLLECTED AND COMMUNICATED.)

THE typhoid epidemic at Belfast shows considerable diminution, although upwards of 90 cases were reported at the last weekly notification.

THE Liverpool Select Vestry have appealed against the order of the Local Government Board to close all public vaccination stations. They ask that they may be kept open on trial for one year.

SURGEON-GENERAL HARVEY, C.B., has been appointed Director-General of the Indian Medical Service and Sanitary Commissioner with the Government of India.

THE Governors of University College, Liverpool, are causing plans to be drawn for the new departments for anatomy, medicine, and surgery, towards the building fund of which they have now a considerable sum of money.

THE question of tuberculosis in milk is being seriously taken up in Islington, and the Public Health Committee of the local vestry have instructed their medical officer of health to submit 200 samples of the milk locally supplied to householders to bacteriological examination.

THE foundation-stone of a Cottage Hospital at Clacton-on-Sea has been laid. The hospital will serve as a Diamond Jubilee memorial. A good site, with southern aspect, has been secured through the generosity of Mr. J. Round, M.P. Half the building is to be erected to commence with, and two wards for four and two beds respectively will be provided. About £850 towards the total estimated cost of £1,700 has been contributed.

THE action taken by the French Minister of War in respect to medical aid for employés has created much indignation. An order had been passed that workers employed in the military workshops at Toulouse should receive free medical aid for themselves and their families, and the Minister of War proceeded to seek the co-operation of local practitioners by advertising for tenders from medical men to attend on the patients at so much per case, preference being given to the lowest tender.

THE decision of the Edinburgh Town Council to erect a new fever hospital on the outskirts of the city has met with much approval. The site chosen at Collinton Mains is a very good one, standing 400 feet above the sea level. Two hundred patients are to be accommodated, and the estimated cost per bed is £360, or £400 including the site.

AT the last quarterly meeting of the Board of Delegates of the Hospital Saturday Fund the report of the Council, which was presented and adopted, showed that the receipts from January 8th to October 15th had amounted to £11,197 0s. 3d., being a decrease as compared with the corresponding period of last year of £1,641 12s. 8d. Of the total the general workshop collection reached £10,803 6s. 7d.; "Hospital Saturday" special collection (part result), £108 7s. 6d.; premises fund, £201 1s.; and miscellaneous, £84 5s. 2d. The expenditure during this period was £1,665 10s. 3d.

THE Devon and Exeter Hospital has now before it the consideration of an offer of the Ockenden Convalescent Home at Torquay. The offer carries conditions. The lady who owns the Home offers to make a donation of £400 with the building, but requires the hospital to provide a matron and undertake that a certain number of ladies resident in Torquay shall be visitors. The committee are not unanimous in thinking that the offer, on account of the conditions attached, will be wholly beneficial, and so the matter is postponed for the present.

THE past year has been one of very marked improvement at the Glasgow Western Infirmary. Three new operating theatres have been added, all of modern construction and arrangement, whilst a fourth operating-room is being refitted. A ward has been arranged with 16 beds for the reception of patients with burns. Twelve additional rooms and a dining-hall have been added to the Nurses' Home. A new laundry, four times the size of the old one, has been erected, and a store-room for patients' clothes provided. The electric light has been laid on everywhere, and extra steam heating apparatus introduced.

THE statement so persistently made by some of the opponents of vaccination that the decline of small-pox during this century is due to improved sanitation has attracted much attention, and has been much relied upon by those outside medical circles to prove that vaccination has had nothing to do with the improved condition of affairs. It is important, then, to observe, as is ably shown by Walter Lloyd in the *Westminster Review*, that if, instead of comparing the present state of affairs with that which held good at the beginning of the century, we enter into a more detailed examination of the different periods, we find that during the middle period the sanitary condition of London underwent great deterioration in consequence of the rapid growth of the population, and that during this period both the general and the zymotic mortality were considerably increased; in fact, the general death-rate became "at least 25 per cent. higher than it was between 1820 and 1830." Yet during this insanitary period the small-pox mortality continued to decline, showing how little insanitation has had to do with the prevalence of the disease.

